# Gender-specific associations of skeletal muscle mass and arterial stiffness among peritoneal dialysis patients

**DOI:** 10.1038/s41598-018-19710-6

**Published:** 2018-01-22

**Authors:** Xinhui Liu, Xunhua Zheng, Chunyan Yi, Juan Wu, Hongjian Ye, Qunying Guo, Xueqing Yu, Xiao Yang

**Affiliations:** 1 Department of Nephrology, The First Affiliated Hospital, Sun Yat-sen University, Guangzhou, Guangdong, 510080 China; 2 Key Laboratory of Nephrology, Ministry of Health and Guangdong Province, Guangzhou, Guangdong, 510080 China; 3 Department of Nephrology, Shenzhen Traditional Chinese Medicine Hospital, Guangzhou University of Chinese Medicine, Shenzhen, Guangdong, 518033 China

## Abstract

Decreased skeletal muscle has been identified as a relevant factor for arterial stiffness but has not been thoroughly investigated in peritoneal dialysis (PD) patients. The aim of this study was to investigate the relationship between skeletal muscle and arterial stiffness in PD patients. A cross-sectional study of 658 prevalent PD patients with a mean brachial-ankle pulse wave velocity (baPWV) of 1714 (±501) cm/s and mean skeletal muscle mass of 26.6 (±5.4) kg was performed. Skeletal muscle mass level was significantly higher in males than in females. When examining skeletal muscle mass as a continuous variable, skeletal muscle mass was significantly associated with baPWV in fully adjusted linear regression models in total patients [standardized coefficients (β), −0.181; 95% confidence interval (95% CI), −0.276 to −0.056; *P* = 0.003] or female patients (β, −0.119; 95% CI, −0.350 to −0.015; *P* = 0.03) but not in male patients (β, −0.117; 95% CI, −0.300 to 0.011; *P* = 0.07). Furthermore, in females, a significant association between the middle or highest tertile of skeletal muscle mass and baPWV was found in fully adjusted models (β, −0.123; 95% CI, −0.204 to −0.008; *P* = 0.03; β, −0.140; 95% CI, −0.228 to −0.016; *P* = 0.02, respectively). In conclusion, decreased skeletal muscle mass was independently associated with increased baPWV in PD patients, and this association was significant in females but not in males.

## Introduction

Peritoneal dialysis (PD) is a well-established modality of renal replacement therapy and is becoming more important in the treatment of patients with end-stage renal disease (ESRD)^1,2^. Cardiovascular disease (CVD) is the leading cause of death in PD patients, according to various national and regional registries^3^. Therefore, proper assessment and treatment of various cardiovascular risk factors are essential parts of the management of PD patients. According to US Renal Data System (USRDS) 2016 annual data report, 53.5% of all ESRD deaths are due to CVD after excluding missing and unknown causes of death. However, only 11.4% of these cases are attributable to acute myocardial infarction and atherosclerotic heart disease, with up to 82.4% deaths being attributed to arrhythmia/cardiac arrest and congestive heart failure (CHF)^4^. Thus structural heart diseases leading to CHF and sudden cardiac death, rather than vasculo-occlusive disease are the leading cause of cardiovascular mortality in ESRD patients. Accumulating evidence suggests that increased arterial stiffness is a major cause of this structural heart disease^5^. Previous longitudinal epidemiological studies have demonstrated the independent predictive value of arterial stiffness for CVD mortality in dialysis patients^6–8^. For this reason, identifying and correcting modifiable factors associated with arterial stiffness will be beneficial to reduce CVD burden in PD patients.

Changes in body composition, such as increased body fat, especially visceral fat, and decreased skeletal muscle, are common among patients with chronic kidney disease (CKD) and are associated with worse physical functioning and mortality^9^. Previous studies have reported that increased visceral fat and reduced skeletal muscle mass are independently associated with increased brachial-ankle pulse wave velocity (baPWV), which is an indicator of arterial stiffness in the general population^10^, the elderly^11^, and type 2 diabetic subjects^12,13^. For PD patients, it was demonstrated that visceral fat level was an independent predictor of PWV in a cross-sectional study^14^. However, the relationship between skeletal muscle mass and arterial stiffness in PD patients remains unknown. More recently, two studies both reported that low skeletal muscle mass level was an independent risk factor for mortality in PD patients^15,16^, which underlined the importance of skeletal muscle mass.

Physiologically, males have significantly higher skeletal muscle mass levels than females. A previous study including healthy middle-aged to elderly persons found that thigh muscle mass was significantly associated with baPWV in men but not in women^17^. Therefore, we hypothesized that there would be a gender-specific association between skeletal muscle mass and baPWV, after which we performed subgroup analyses to assess whether this association differed in male and female PD patients.

## Results

### Patient characteristics

**Figure 1:**
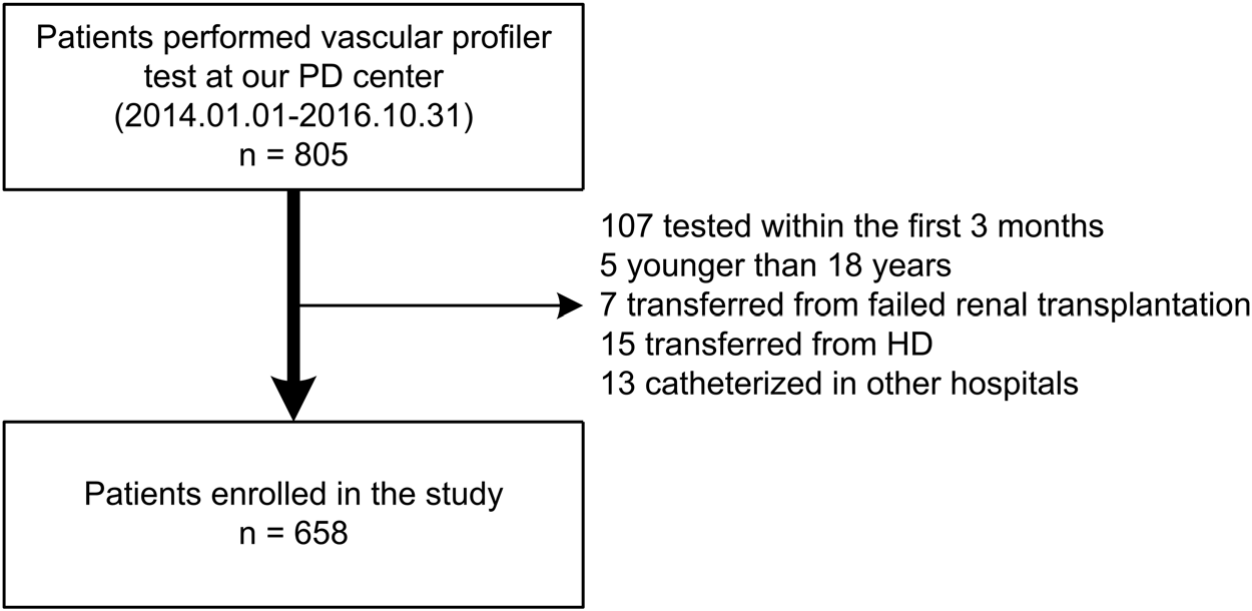
The flow chart shows how patients were selected for the present study. HD, hemodialysis; PD, peritoneal dialysis.

In total, 805 continuous ambulatory PD (CAPD) patients underwent a vascular profiler test at our PD center from January 1, 2014, to October 31, 2016, of whom 107 patients were on PD less than 3 months, 5 patients were younger than 18 years, 7 patients were transferred from failed renal transplantation, 15 patients were transferred from permanent hemodialysis, and 13 patients were catheterized in another hospital. The remaining 658 patients were enrolled in this study (Fig. 1). The mean (±SD) age was 46.7 ± 13.9 years, the median PD vintage was 32 months (interquartile range 11 to 57 months), 56.4% of patients were men, and 15.8% of patients were diabetic. The primary cause of ESRD was chronic glomerulonephritis (68.4%), followed by diabetic nephropathy (12.8%) and hypertension (8.4%). The average baPWV value was 1714 cm/s, and the mean (±SD) skeletal muscle mass was 26.6 ± 5.4 kg (Table 1). All patients received CAPD treatment. Conventional PD solutions (Dianeal 1.5%, 2.5% or 4.25% dextrose; Baxter Healthcare, Guangzhou, China), Y sets, and twin bag systems were used in all PD patients. Physiologic calcium peritoneal dialysate (Ca^2+^ concentration = 1.25 mmol/L) was prescribed to all patients in our PD center. The management of the PD patients was implemented based on the guidelines from the International Society for Peritoneal Dialysis and patient characteristics in our PD center^1,18^. As expected, the distribution of skeletal muscle mass significantly differed between genders, with men showing higher skeletal muscle mass than women (see Supplementary Fig. S1, Table 1). In addition, men showed decreasing levels of skeletal muscle mass with age, whereas women did not. Nevertheless, at each 10-year age group, men displayed higher skeletal muscle mass than did women (see Supplementary Fig. S2). Male patients also had higher high sensitivity C-reactive protein (HsCRP), serum phosphorus, and serum uric acid but lower visceral fat area, calcium, triglyceride, total, low-density lipoprotein, and high-density lipoprotein cholesterol levels compared to female patients. Men also more frequently took calcium channel blockers and phosphate binders compared to women. There was no significant difference between genders in age, body mass index, comorbidity score, PD vintage, and baPWV values (Table 1).

**Table 1:** Characteristics of the study population by gender.

Variable^a^	Total (n = 658)	Male (n = 371)	Female (n = 287)	*P* Value^b^
Demographics				
Age, yr	46.7 ± 13.9	46.1 ± 13.7	47.5 ± 14.0	0.21
Body mass index, kg/m^2^	22.4 ± 3.3	22.6 ± 3.1	22.2 ± 3.7	0.17
Diabetes, *n* (%)	104 (15.8)	71 (19.1)	33 (11.5)	**0.008**
CVD, *n* (%)	280 (42.6)	171 (46.1)	109 (38.0)	**0.04**
Primary kidney disease, *n* (%)				
Chronic glomerulonephritis	450 (68.4)	244 (65.8)	206 (71.8)	0.10
Diabetic nephropathy	84 (12.8)	57 (15.4)	27 (9.4)	**0.02**
Hypertension	55 (8.4)	36 (9.7)	19 (6.6)	0.16
Other	69 (10.5)	34 (9.2)	35 (12.2)	0.21
Charlson comorbidity score	2 (2–3)	2 (2–4)	2 (2–3)	0.20
PD information				
PD vintage, mo	32 (11–57)	28 (11–54)	36 (11–61)	0.09
PD solution glucose concentration, *n* (%)				
1.5%	636 (96.7)	359 (99.4)	277 (98.9)	0.46
2.5%	307 (46.7)	170 (47.1)	137 (48.9)	0.64
4.25%	23 (3.5)	11 (3.0)	12 (4.3)	0.40
PET category, n (%)				
Low	12 (2.4)	5 (1.7)	7 (3.4)	0.24
Low average	155 (31.3)	79 (27.4)	76 (36.7)	**0.03**
High average	247 (49.9)	147 (51.0)	100 (48.3)	0.55
High	81 (16.4)	57 (19.8)	24 (11.6)	**0.02**
Kt/V	2.3 ± 0.9	2.1 ± 0.9	2.5 ± 0.7	**<0.001**
Residual urine volume, mL	400 (50–885)	450 (50–1000)	400 (50–800)	0.41
mGFR, mL/min/1.73 m^2^	2.90 (0.98–4.67)	2.79 (0.49–4.21)	3.49 (1.92–5.08)	0.15
Laboratory parameters				
Hemoglobin, g/dL	11.1 ± 2.0	11.2 ± 2.0	11.0 ± 2.0	0.21
N/L	3.28 (2.48–4.35)	3.27 (2.47–4.35)	3.35 (2.54–4.35)	0.54
HsCRP, mg/L	1.52 (0.60–4.58)	1.67 (0.71–4.58)	1.41 (0.51–4.64)	**0.04**
ALP, U/L	91 (70–119)	90 (71–117)	91 (69–126)	0.72
Serum albumin, g/dL	3.66 ± 0.46	3.69 ± 0.50	3.62 ± 0.41	0.06
Serum prealbumin, mg/L	385 (323–455)	394 (331–462)	369 (320–442)	0.06
Corrected calcium, mg/dL	9.56 ± 0.80	9.49 ± 0.81	9.65 ± 0.78	**0.009**
Serum phosphorus, mg/dL	5.10 ± 1.54	5.23 ± 1.55	4.94 ± 1.52	**0.02**
Total cholesterol, mg/dL	193 (166–224)	182 (159–209)	205 (178–240)	**<0.001**
Triglyceride, mg/dL	132 (92–192)	121 (88–175)	151 (97–219)	**<0.001**
HDL-C, mg/dL	45 (38–54)	43 (36–50)	49 (40–56)	**<0.001**
LDL-C, mg/dL	121 (103–146)	116 (98–137)	128 (110–157)	**<0.001**
Serum urea nitrogen, mg/dL	51 ± 16	55 ± 15	47 ± 15	**<0.001**
Serum creatinine, mg/dL	11.1 (8.7–14.0)	12.8 (9.5–15.1)	10.0 (7.9–11.9)	**<0.001**
Uric acid, mg/dL	6.80 ± 1.29	6.95 ± 1.32	6.59 ± 1.24	**<0.001**
iPTH, pg/mL	302 (155–544)	309 (162–517)	291 (142–566)	0.71
BIA analysis				
Waist circumference, cm	87 ± 9	88 ± 9	85 ± 10	**<0.001**
Visceral fat area, cm^2^	43.9 (5.0–75.9)	36.1 (5.0–68.5)	49.7 (12.4–82.2)	**0.01**
Skeletal muscle mass, kg	26.6 ± 5.4	30.1 ± 4.2	22.1 ± 3.1	**<0.001**
Vascular profiler data				
Heart rate, bpm	78 ± 20	77 ± 18	80 ± 21	0.07
Systolic pressure, mmHg	147 ± 22	147 ± 21	147 ± 23	0.81
Mean arterial pressure, mmHg	116 ± 18	116 ± 17	115 ± 18	0.76
Diastolic pressure, mmHg	91 ± 14	91 ± 14	89 ± 14	0.06
Pulse pressure, mmHg	56 ± 14	56 ± 13	57 ± 15	0.15
baPWV, cm/s	1714 ± 501	1717 ± 502	1709 ± 501	0.84
Medications				
Calcium channel blocker use, *n* (%)	502 (76.3)	297 (82.0)	205 (73.2)	**0.007**
ACEI and/or ARB use, *n* (%)	448 (68.1)	248 (69.5)	200 (71.4)	0.59
*β*-blocker use, *n* (%)	383 (58.2)	224 (62.6)	159 (57.6)	0.21
Diuretic use, *n* (%)	61 (9.3)	36 (10.2)	25 (9.0)	0.62
Phosphate binder use, *n* (%)	264 (40.1)	171 (47.6)	93 (33.5)	**<0.001**
Erythropoietin use, *n* (%)	579 (88.0)	318 (88.1)	261 (93.2)	**0.03**
Lipid-lowering agent use, *n* (%)	132 (20.1)	65 (18.2)	67 (24.1)	0.07
Antiplatelet agent use, *n* (%)	132 (20.1)	71 (19.9)	61 (22.0)	0.52

^a^Data were presented as frequency (percentage) for categorical variables, mean ± SD or median (IQR) for continuous variables.

^b^*P* value represents the comparison between male and female PD patients.

Abbreviations: ACEI, angiotensin converting enzyme inhibitor; ALP, alkaline phosphatase; ARB, angiotensin II receptor blocker; baPWV, brachial-ankle pulse wave velocity; BIA, bioelectrical impedance analysis; CVD, cardiovascular disease; HDL-C, high-density lipoprotein cholesterol; HsCRP, high sensitivity C-reactive protein; iPTH, intact parathyroid hormone; LDL-C, low-density lipoprotein cholesterol; mGFR, measured glomerular filtration rate; N/L, neutrophil to lymphocyte ratio; PET, peritoneal equilibration test; PD, peritoneal dialysis.

### Correlation of skeletal muscle mass with baPWV

Correlation analyses in all patients indicated that baPWV level was positively correlated with age, diabetes, comorbidity score, PD vintage, neutrophil-to-lymphocyte ratio (N/L), HsCRP, alkaline phosphatase, total cholesterol, low-density lipoprotein cholesterol, triglyceride, blood pressure, and visceral fat area, while negatively correlated with serum albumin, serum prealbumin, and skeletal muscle mass (*P* < 0.05) (Table 2). Both male and female patients showed a significant correlation between skeletal muscle mass and baPWV.

**Table 2:** Correlation between baPWV and related parameters.

Variable	Total		Male		Female	
	Coefficient	*P* Value	Coefficient	*P* Value	Coefficient	*P* Value
Age	0.406	**<0.001**	0.409	**<0.001**	0.406	**<0.001**
Gender	0.020	0.60	—	—	—	—
Body mass index	−0.059	0.13	−0.056	0.28	−0.064	0.28
Waist circumference	0.071	**0.07**	0.106	**0.04**	0.028	0.64
Diabetes	0.186	**<0.001**	0.183	**<0.001**	0.191	**0.001**
CVD	0.062	0.11	0.072	0.17	0.051	0.39
Charlson comorbidity score	0.421	**<0.001**	0.412	**<0.001**	0.432	**<0.001**
PD vintage	0.172	**<0.001**	0.159	**0.002**	0.192	**0.001**
mGFR	−0.118	0.20	−0.090	0.43	−0.222	0.14
N/L	0.142	**<0.001**	0.194	**<0.001**	0.078	0.19
Hemoglobin	0.032	0.41	0.136	**0.009**	−0.103	0.08
HsCRP	0.178	**<0.001**	0.180	**0.001**	0.177	**0.003**
ALP	0.142	**<0.001**	0.173	**0.001**	0.112	0.06
Serum albumin	−0.205	**<0.001**	−0.183	**<0.001**	−0.246	**<0.001**
Serum prealbumin	−0.102	**0.01**	−0.141	**0.008**	−0.067	0.28
Corrected calcium	0.011	0.78	−0.007	0.89	0.037	0.53
Serum phosphorus	0.021	0.59	0.066	0.21	−0.039	0.51
Total cholesterol	0.102	**0.01**	0.120	**0.02**	0.111	0.06
Triglyceride	0.108	**0.006**	0.105	**0.05**	0.123	**0.04**
HDL-C	−0.031	0.43	−0.037	0.49	−0.001	0.98
LDL-C	0.103	**0.009**	0.108	**0.04**	0.117	**0.05**
iPTH	0.019	0.63	0.081	0.13	−0.056	0.35
Visceral fat area	0.216	**<0.001**	0.231	**<0.001**	0.190	**0.003**
Skeletal muscle mass	−0.109	**0.01**	−0.192	**0.001**	−0.144	**0.03**
Systolic pressure	0.379	**<0.001**	0.335	**<0.001**	0.432	**<0.001**
Mean arterial pressure	0.326	**<0.001**	0.282	**<0.001**	0.379	**<0.001**
Diastolic pressure	0.255	**<0.001**	0.218	**<0.001**	0.303	**<0.001**
Pulse pressure	0.352	**<0.001**	0.327	**<0.001**	0.386	**<0.001**

Abbreviations: ALP, alkaline phosphatase; CVD, cardiovascular disease; HDL-C, high-density lipoprotein cholesterol; HsCRP, high sensitivity C-reactive protein; iPTH, intact parathyroid hormone; LDL-C, low-density lipoprotein cholesterol; mGFR, measured glomerular filtration rate; N/L, neutrophil to lymphocyte ratio; PD, peritoneal dialysis.

### Association of skeletal muscle mass with baPWV

The associations between skeletal muscle mass and baPWV were evaluated in total, male, and female patients and are summarized in Table 3. First, we tested the association between the interaction of sex with skeletal muscle mass and baPWV and observed a significant association [standardized coefficients (β), −0.092; 95% confidence interval (95% CI), −0.154 to −0.017; *P* = 0.02]. When skeletal muscle mass was examined as a continuous variable, skeletal muscle mass was significantly associated with baPWV in fully adjusted models in total (β, −0.181; 95% CI, −0.276 to −0.056; *P* = 0.003) or female (β, −0.119; 95% CI, −0.350 to −0.015; *P* = 0.03) patients but not in male patients (β, −0.117; 95% CI, −0.300 to 0.011; *P* = 0.07). To further dissect the differential association between skeletal muscle mass and baPWV by gender, we stratified patients in gender-specific tertiles of skeletal muscle mass. In females, a significant association between the middle or highest tertile of skeletal muscle mass and baPWV was found in fully adjusted models [β, −0.123; 95% CI, −0.204 to −0.008; *P* = 0.03; β, −0.140; 95% CI, −0.228 to −0.016; *P* = 0.02, respectively]. In contrast, in males, only the middle tertile of skeletal muscle mass was significantly associated with baPWV (β, −0.121; 95% CI, −0.226 to −0.007; *P* = 0.04).

**Table 3:** Associations between skeletal muscle mass and baPWV in total, male, and female patients by multiple linear regression analysis.

Variables	Model 1^a^		Model 2^b,d^		Model 3^c,d^	
	β (95% CI)	*P* Value	β (95% CI)	*P* Value	β (95% CI)	*P* Value
Sex × skeletal muscle mass	−0.110 (−0.174 to −0.023)	**0.01**	−0.103 (−0.160 to −0.029)	**0.005**	−0.092 (−0.154 to −0.017)	**0.02**
Continuous skeletal muscle mass						
Total	−0.109 (−0.173 to −0.022)	**0.01**	−0.206 (−0.293 to −0.079)	**0.001**	−0.181 (−0.276 to −0.056)	**0.003**
Male	−0.192 (−0.369 to −0.097)	**0.001**	−0.120 (−0.298 to 0.003)	0.06	−0.117 (−0.300 to 0.011)	0.07
Female	−0.144 (−0.397 to −0.025)	**0.03**	−0.128 (−0.356 to −0.033)	**0.02**	−0.119 (−0.350 to −0.015)	**0.03**
Skeletal muscle mass tertiles						
Total tertile 1	Ref		Ref		Ref	
Total tertile 2	0.037 (−0.054 to 0.121)	0.46	0.006 (−0.087 to 0.097)	0.91	0.013 (−0.081 to 0.104)	0.80
Total tertile 3	−0.071 (−0.151 to 0.024)	0.15	−0.096 (−0.207 to 0.032)	0.15	−0.081 (−0.196 to 0.046)	0.23
Male tertile 1	Ref		Ref		Ref	
Male tertile 2	−0.197 (−0.306 to −0.063)	**0.003**	−0.105 (−0.210 to 0.011)	0.08	−0.121 (−0.226 to −0.007)	**0.04**
Male tertile 3	−0.192 (−0.301 to −0.059)	**0.004**	−0.105 (−0.225 to 0.025)	0.12	−0.106 (−0.229 to 0.025)	0.12
Female tertile 1	Ref		Ref		Ref	
Female tertile 2	−0.223 (−0.310 to −0.065)	**0.003**	−0.150 (−0.223 to −0.034)	**0.008**	−0.123 (−0.204 to −0.008)	**0.03**
Female tertile 3	−0.158 (−0.255 to −0.011)	**0.03**	−0.150 (−0.234 to −0.027)	**0.01**	−0.140 (−0.228 to −0.016)	**0.02**

^a^Model 1: Unadjusted.

^b^Model 2: Adjusted for age, gender, body mass index, heart rate, diabetes, cardiovascular disease, PD vintage, mean arterial pressure, and residual urine volume.

^c^Model 3: Model 2 + adjusted for high sensitivity C-reactive protein, serum albumin, total cholesterol, and intact parathyroid hormone.

^d^Did not adjust gender while analyzing sex × skeletal muscle mass, male or female PD patients.

Abbreviations: 95% CI, 95% confidence interval.

To explain the gender difference, sensitivity analyses were performed. We conducted multiple linear regression analyses in each tertile of patients separately. The results showed that skeletal muscle mass was significantly associated with baPWV in a fully adjusted model only in the lowest tertile patients (≤24 kg) (β, −0.188; 95% CI, −0.675 to −0.160; *P* = 0.002) but not in the middle tertile patients (24–29 kg) (β, −0.048; 95% CI, −0.818 to 0.402; *P* = 0.50) or the highest tertile patients (>29 kg) (β, −0.111, 95% CI, −0.267 to 0.038, *P* = 0.14) (see Supplementary Table S1). Further analysis indicated that 93.3% of patients in the lowest tertile were women.

## Discussion

In this cross-sectional study, we disclosed a significant and independent association between decreased skeletal muscle mass and increased baPWV in PD patients. This association was significant in females but not in males, which may be explained by the higher skeletal muscle mass level in males compared to females.

The arterial system of ESRD patients undergoes remodeling that is characterized by dilation and arterial intima-media hypertrophy. The alterations of intrinsic properties of arterial wall materials will increase arterial stiffness^19^. Aortic PWV has been recommended as the gold-standard measure of arterial stiffness^20^. Aortic PWV is a powerful independent predictor of all-cause mortality and cardiovascular events in ESRD, hypertensive subjects, the elderly, diabetic subjects and the general population^21^. Previous studies have identified some risk factors associated with aortic PWV in PD patients, such as malnutrition^22^, peritoneal transport status^23^, residual renal function^24^, and inflammation^25^. In the present study, we first showed that skeletal muscle mass was an independent determinant of baPWV in PD patients. This result echoes previous studies in various populations^10–13^ and hemodialysis patients^26^. Patients with chronic kidney disease are potentially at greater risk of muscle wasting because of increased urinary protein losses and reduced dietary protein intake^27^, and PD patients may also have protein and amino acid losses in the dialysate, especially in higher peritoneal transporters^28,29^. Recent studies have shown that muscle loss was prevalent in PD patients^30^, while shortage of skeletal muscle was an independent risk factor for mortality in PD patients^15,16^. Skeletal muscle may be viewed as an endocrine organ that releases numerous factors with the potential to influence vascular tone. Greater lean soft tissue mass attenuates age-related increases in arterial stiffness^31,32^.

Of note, gender-specific associations were found between skeletal muscle mass and baPWV in our study. By sensitivity analysis, we believe that lower skeletal muscle mass levels were generally reached by women compared with men, which may explain this gender difference. The gender-specific tertile analysis may suggest a threshold effect in males and females. There are several potential mechanisms why skeletal muscle mass decline may be associated with increased arterial stiffness in PD patients. First, since skeletal muscle mass was well correlated with serum prealbumin, low skeletal muscle mass may reflect a poor nutritional status, which is a predefined risk factor of arterial stiffness^33^. Second, skeletal muscle is the main site for insulin-mediated glucose disposal, and low skeletal muscle mass may be associated with insulin resistance^34^. Third, decreased skeletal muscle mass will result in low physical activity, which may lead to the promotion of arteriosclerosis^35^.

There are several limitations in the present study. First, this is a single-center study, and thus center-specific effects cannot be excluded. Second, given the cross-sectional nature of our study, we established associations but not causal relationships. Third, skeletal muscle mass of PD patients in this study was measured using BIA, which is considered to be not “ideal” but a feasible method for body composition assessment and is widely used in dialysis patients^36,37^. The usefulness of BIA in measuring body composition was cross-validated against dual-energy X-ray absorptiometry^38,39^, which is regarded as the most practical means of obtaining an accurate assessment of fat-free mass and fat mass in dialysis patients^40^. Finally, the potential co-pathogenesis behind skeletal muscle decline and arterial stiffness was not fully investigated. Thus, the effect of residual confounding cannot be eliminated completely.

In conclusion, our data indicated that skeletal muscle decline was independently associated with increased baPWV in PD patients. Further prospective studies will be needed to investigate the predicative role of skeletal muscle mass in cardiovascular events and mortality in PD patients.

## Methods

### Study population and data collection

We studied all CAPD patients who performed a vascular profiler test at the PD center of The First Affiliated Hospital, Sun Yat-sen University, Guangzhou, China from January 1, 2014 to October 31, 2016. Inclusion criteria were age ≥18years at the time of vascular profiler test and survival for at least 90 days from the first PD therapy. The patients who were catheterized in other hospitals, transferred from permanent hemodialysis (≥3 months) or failed renal transplantation were excluded in this study. The study was conducted in compliance with the ethical principles of the Helsinki Declaration and approved by the Human Ethics Committees of Sun Yat-sen University. Written informed consent was obtained from all participants.

Demographic data included age, gender, body mass index (BMI), primary cause of ESRD and presence of diabetes and CVD. Clinical and biochemical data included PD vintage, Kt/V, residual urine volume, measured glomerular filtration rate (mGFR) calculated from 24 h urine collections and indexed for the body surface area, N/L, hemoglobin, serum albumin, prealbumin, HsCRP, serum lipid profile, serum calcium, phosphorus, intact parathyroid hormone (iPTH), alkaline phosphatase, and uric acid. Medication information was also collected. The comorbidity score was determined according to the Charlson Comorbidity Index, which is one of the most commonly used comorbidity models^41^.

### Body composition measurement

Body composition was measured by multi-frequency bioelectrical impedance analysis (BIA) InBody 720 (Biospace, Seoul, Korea). InBody 720 uses state-of-the-art technology and an 8-point tactile electrode system that measures the total and segmental impedance and phase angle of alternating electric current at six different frequencies (1 kHz, 5 kHz, 50 kHz, 250 kHz, 500 kHz, and 1000 kHz). The operation of InBody 720 was described in detail in our previous publication^42^. All the body composition data were performed in the instrument using internal software.

### Brachial-ankle PWV measurement

The values of baPWV were measured using a noninvasive automatic vascular profiler device BP-203RPE III (Omron, Japan) as previously described^43,44^. Briefly, following 10 minutes of rest in the supine position, four oscillometric cuffs were applied to bilateral brachia and ankles, and electrocardiograph electrodes were placed on bilateral wrists. The transmission distance from the brachium to ankle was calculated according to height of the patient. The path length from the suprasternal notch to the brachium (Lb) was obtained using the following equation: Lb = 0.2195 × height (cm) − 2.0734. The path length from the suprasternal notch to the ankle (La) was obtained using the following equation: La = 0.8129 × height (cm) + 12.328. The baPWV was calculated according to the following formula: baPWV = (La − Lb)/Tba, where Tba was the time interval between the initial increase in brachial and ankle waveforms. The validation of this automatic device and its reproducibility have been previously published^45^. After obtaining bilateral baPWV values, the average value was used for analysis. Heart rate and systolic and diastolic blood pressure were also obtained by the same device. The average values of systolic and diastolic blood pressure of bilateral brachia were used for analysis.

### Statistical analysis

We compared demographic, clinical and laboratory parameters, body composition data, vascular profiler data, and medications information between male and female PD patients. The results were expressed as frequencies and percentages for categorical variables, means and standard deviations for normally distributed continuous variables, and medians and interquartile ranges for continuous variables not normally distributed. Chi-squared test, independent-samples *t*-test, and Mann-Whitney U test were used to test for differences in categorical or continuous factors between groups. The correlations between baPWV and other variables were assessed by Pearson correlation test for normally distributed variables or Spearman rank correlation test for non-parametric variables. The associations between skeletal muscle mass and baPWV were examined by multiple linear regression models in total, male, and female patients. Moreover, the interaction between sex and skeletal muscle mass was examined by performing a formal test of interaction. Variables with *P* < 0.05 in the univariate analysis were picked into multivariate adjusted model according to following principles: presenting clinical relevance and avoiding multicollinearity. Since previous studies have demonstrated that heart rate is an important confounder of PWV^46^, we forced heart rate into models. These covariates of malnutrition, peritoneal transport status, residual renal function, and inflammation were chosen based on their statistical associations with baPWV in univariate model (*P* < 0.05). Thus, the final full model included age, gender, BMI, heart rate, diabetes, CVD, PD vintage, mean arterial pressure, residual urine volume, HsCRP, serum albumin, total cholesterol, and iPTH. To further assess the different associations with baPWV at different skeletal muscle mass levels, gender-specific tertiles of skeletal muscle mass were also built using the lowest gender-specific skeletal muscle mass tertile as the reference group. The complete case analysis was used to handle missing data. The results were expressed as β and 95% CI. All analyses in the present study were conducted using SPSS version 16.0 (SPSS Inc., Chicago, IL, USA). A value of *P* < 0.05 was considered statistically significant.

## Electronic supplementary material

Supplementary Information
