# Supplementary material for: Gender-specific associations of skeletal muscle mass and arterial stiffness among peritoneal dialysis patients
Source: Sci Rep. 2018 Jan 22;8:1351. doi: 10.1038/s41598-018-19710-6 (PMC5778131; doi:10.1038/s41598-018-19710-6)

Gender-specific associations of skeletal muscle mass and arterial stiffness among peritoneal dialysis patients

Xinhui Liu,[1,2,3#] Xunhua Zheng,[1,2#] Chunyan Yi,[1,2] Juan Wu,[1,2] Hongjian Ye,[1,2] Qunying Guo,[1,2] Xueqing Yu,[1,2] Xiao Yang [1,2*]

[1] Department of Nephrology, The First Affiliated Hospital, Sun Yat-sen University, Guangzhou, Guangdong 510080, China

[2] Key Laboratory of Nephrology, Ministry of Health and Guangdong Province, Guangzhou, Guangdong 510080, China

[3] Department of Nephrology, Shenzhen Traditional Chinese Medicine Hospital, Guangzhou University of Chinese Medicine, Shenzhen, Guangdong 518033, China

# Xinhui Liu and Xunhua Zheng contributed equally to this study.

* Corresponding Author: Prof. Xiao Yang, MD & PhD

Full address: Department of Nephrology, The First Affiliated Hospital, Sun Yat-sen University, Guangzhou, Guangdong 510080, China

Tel number: 86-20-87755766-8843

Fax number: 86-20-87766335

Email: yangxsysu@126.com

Supplementary Table S1. Associations between different tertile of skeletal muscle mass and baPWV in total patients by fully adjusted linear regression model

Skeletal muscle mass as continuous variable [a]	β (95% CI)	*P* Value
Tertile 1 (≤24 kg)	-0.188 (-0.675 to -0.160)	**0.002**
Tertile 2 (24-29 kg)	-0.048 (-0.818 to 0.402)	0.50
Tertile 3 (>29 kg)	-0.111 (-0.267 to 0.038)	0.14

[a]: Adjusted for age, gender, body mass index, heart rate, diabetes, cardiovascular disease, PD vintage, mean arterial pressure, residual urine volume, high sensitivity C-reactive protein, serum albumin, total cholesterol, and intact parathyroid hormone.

Abbreviations: 95% CI, 95% confidence interval.

Supplementary Figure S1. The distribution of skeletal muscle mass by gender.

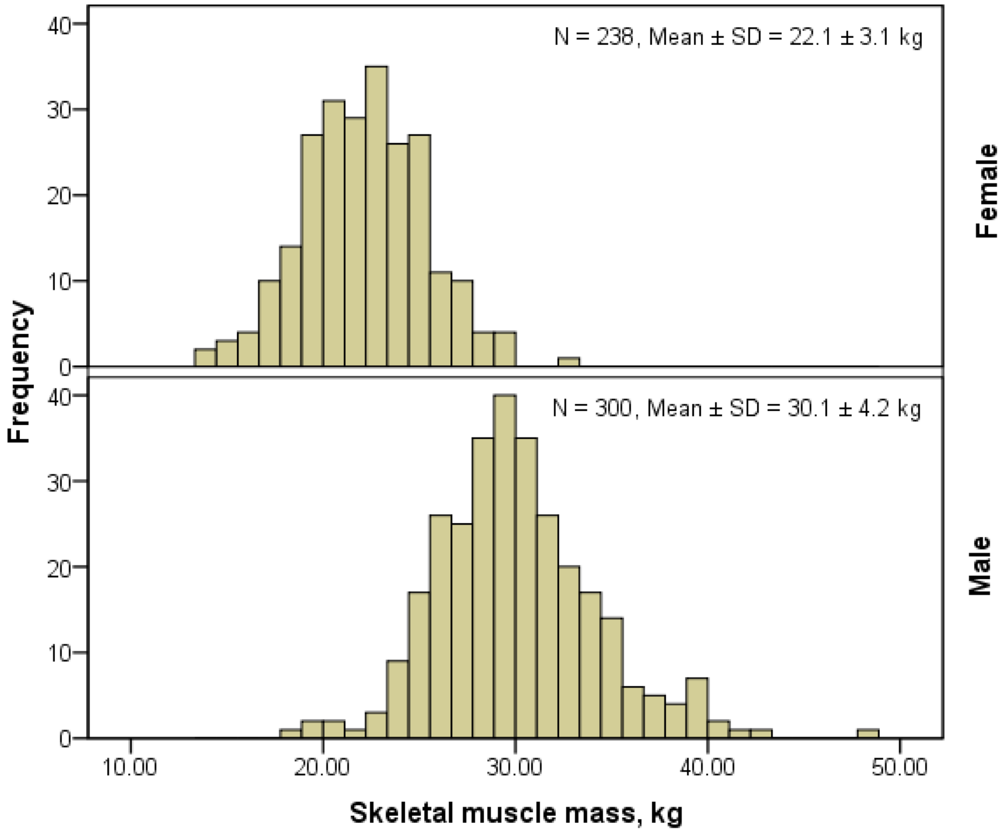

Supplementary Figure S2. The level of skeletal muscle mass at each 10-year age group by gender.

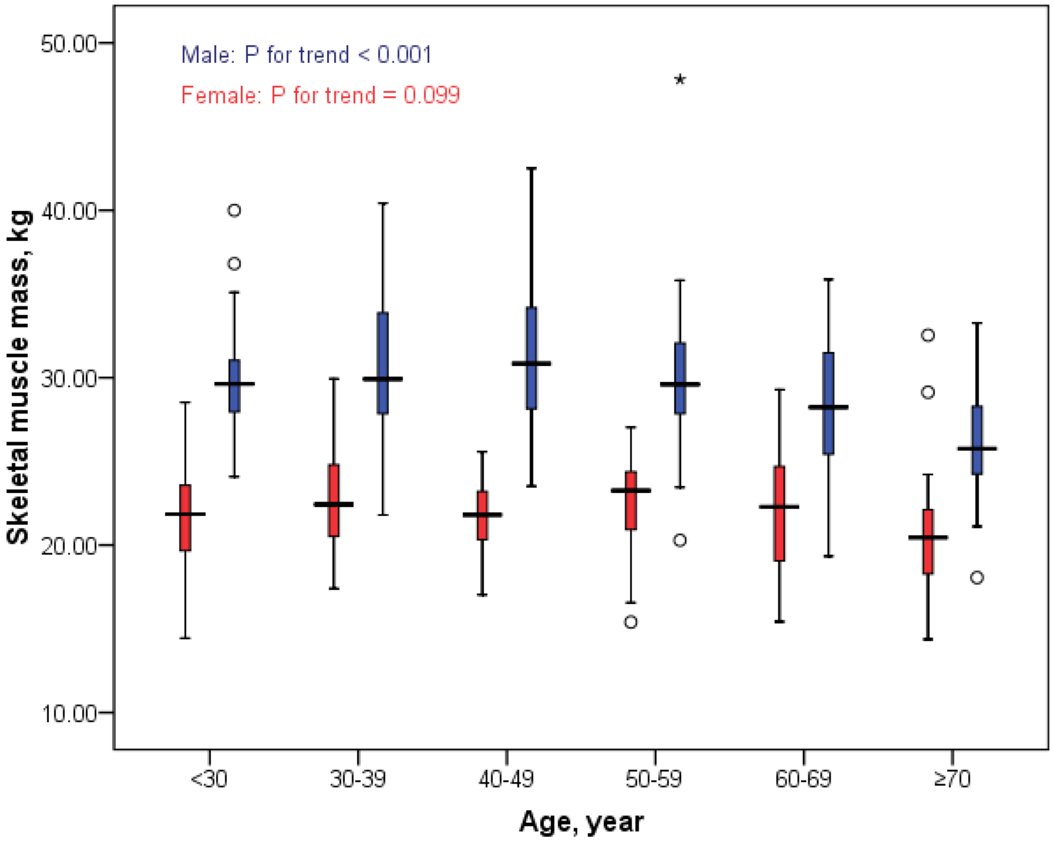

Supplement: Supplementary file 1 — Supplementary Information [file 41598_2018_19710_MOESM1_ESM.pdf]
